# The effect of botulinum toxin-A injection into the masseter muscles on prevention of plate fracture and post-operative relapse in patients receiving orthognathic surgery

**DOI:** 10.1186/s40902-018-0174-0

**Published:** 2018-11-25

**Authors:** Sung-Ho Shin, Yei-Jin Kang, Seong-Gon Kim

**Affiliations:** Department of Oral and Maxillofacial Surgery, College of Dentistry, Gangneung-Wonju National University, Jukheon gil 7, Gangneung, Gangwondo 25457 Republic of Korea

**Keywords:** Botulinum toxins, Type A, Fracture fixation, Internal, Osteotomy, Sagittal split ramus, Postoperative complications

## Abstract

**Background:**

Botulinum toxin-A (BTX-A) injection into muscle reduces muscular power and may prevent post-operative complication after orthognathic surgery. The purpose of this study was (1) to evaluate BTX-A injection into the masseter muscle on the prevention of plate fracture and (2) to compare post-operative relapse between the BTX-A injection group and the no injection group.

**Methods:**

Sixteen patients were included in this study. Eight patients received BTX-A injection bilaterally, and eight patients served as control. All patients received bilateral sagittal split ramus osteotomy for the mandibular setback and additional surgery, such as LeFort I osteotomy or genioplasty. Post-operative plate fracture was recorded. SNB angle, mandibular plane angle, and gonial angle were used for post-operative relapse.

**Results:**

Total number of fractured plates in patients was 2 out of 16 plates in the BTX-A injection group and that was 8 out of 16 plates in the no treatment group (*P* = 0.031). However, there were no significant differences in post-operative changes in SNB angle, mandibular plane angle, and gonial angle between groups (*P* > 0.05).

**Conclusions:**

BTX-A injection into the masseter muscle could reduce the incidence of plate fracture.

## Background

Botulinum toxin-A (BTX-A) is produced by *Clostridium botulinum* bacterium and has been used for esthetics and therapeutic purposes [1]. Once injected to the muscle, BTX-A binds to the presynaptic terminal end and releases acetylcholine. With these reactions, BTX-A can reduce the activity of muscles effectively and safely [2]. The most common application of BTX-A in the field of maxillofacial plastic and reconstructive surgery is wrinkle removal in facial skin [3]. Recently, BTX-A has been used for therapeutic purposes in temporomandibular disorder [4]. As BTX-A injection induces muscular weakness, BTX-A injection into the anterior belly of the digastric muscle can correct open bite which is caused by bilateral mandibular angle fracture [5].

Orthognathic surgery is a surgical procedure for the correction of dentofacial deformities [6]. Post-operative relapse is a tendency of the facial skeleton to move from its pre-operative anatomical position [7]. Many kinds of relapse mechanisms have been introduced [8]. The first is improper bony interference after surgery on sagittal split ramus osteotomy (SSRO) techniques [9]. This unavoidable bony interference leads to displacement of the proximal segment and results in early relapse [10]. The second is related to the condyle. The improper condyle position or excessive torque to the condyle results in relapse [8]. Condyle malposition after surgery, also known as condyle sag, is one of the main causes of early relapse in orthognathic surgery patients [11]. Excessive torque on the condyle is the cause of relapse and long-term temporomandibular disorder in orthognathic patients [12]. To prevent these problems, semi-rigid fixation and condylar repositioning systems have been introduced [13]. The third relapse mechanism involves paramandibular soft tissues, including muscles [8]. To prevent this problem, myotomy has been considered [14]. However, this technique is somewhat invasive and results in the discomfort of patients, including swelling and bleeding after surgery [14].

A single four-hole plate with mono-cortical fixation can be achieved via an intra-oral approach. Using contra-angle, drilling and screwing can be done via an intra-oral approach. Two four-hole plates are more stable than a single four-hole plate for the fixation of the mandibular ramus [15]. The stress on the condyle is less in a single four-hole plate system than two two-hole plate systems [16]. As absorbable plates are weaker than titanium plates, a longer intermaxillary fixation period is required [17]. Intermaxillary fixation for a long period of time is uncomfortable for the patient [18].

To reduce the incidence of plate fracture, reduction of masticatory muscle power might be helpful. The proximal segment of the mandible provides the attachment for the masseter muscle, temporal muscle, and external pterygoid muscle. Among them, the masseter muscle is used for the injection site of BTX-A. BTX-A injection into the masseter muscle reduces its muscular power [19]. Because muscle is one of the contributing factors for post-operative relapse [20], BTX-A injection may reduce post-operative relapse. The purpose of this preliminary study was (1) to evaluate BTX-A injection into the masseter muscle on the prevention of plate fracture and (2) to compare post-operative relapse between the BTX-A injection group and the no injection group.

## Methods

### Ethical approval

This retrospective study was approved by the Institutional Review Board of Gangneung-Wonju National University Dental Hospital (IRB 2018-004).

### Patients

The clinical records of patients who visited Gangneung-Wonju National University Dental Hospital for orthognathic surgery from January 1, 2012, to March 1, 2018, were used for the evaluation. Inclusion criteria were as follows: (1) patients received bilateral sagittal split ramus osteotomy (BSSRO) for mandibular surgery, (2) patients received single four-hole extended titanium mini-plate for the fixation of mandibular ramus on each side, and (3) patients having skeletal class III malocclusion. Exclusion criteria were as follows: (1) congenital deformity, such as like cleft lip and palate or syndromic patients; and (2) patients who received the mandibular advancement surgery. Clinical records and radiographs of 16 patients were used in this study. The mean age of patients was 22.25 years (range 18–34 years). Eight patients were treated with orthognathic surgery only, and another eight patients received additional BTX-A injection into both masseter muscles immediately after orthognathic surgery.

### Surgical techniques

The routine mandibular orthognathic surgery with the short lingual technique was performed for the patients. For the fixation of the ramus to the distal segment of the mandible, single four-hole plates with four pieces of 6-mm mini-screws were used with a rectangular screwing device. All procedures were performed and done intra-orally without a transbuccal approach. As additional surgery, some patients received Le Fort I osteotomy and/ or genioplasty (Table 1). In the BTX-A injection group, the BTX-A was injected immediately after the surgery. Five units of BTX-A was injected into five sites on each masseter muscle. Accordingly, 25 units of BTX-A was given to each masseter muscle. Intermaxillary fixation after surgery was done for 1 week, and then patients were allowed to open their mouths with the guidance of rubber rings. Post-operative follow-up was done at 2 and 6 months postoperatively. Any complication during follow-up was recorded.

**Table 1:** Summary of patients

		No treatment	BTX-A injection
Age (years)		24.0 ± 6.1	21.3 ± 2.4
Sex	Female	4	2
	Male	4	6
Amount of setback (mm)		7.61 ± 4.04	5.70 ± 2.44
Additional treatment	LeFort I osteotomy	5	3
	Genioplasty	3	5
	Angle reduction	0	1
	Lower border reduction	1	0

### Radiographic analysis

The postoperative panorama view was used for counting the number of fractured plates. On the postoperative lateral cephalometric radiographs, the change of SNB angle, mandibular plane angle, and gonial angle was measured. The difference of measurement between immediately after operation and 6 months after operation was considered as the change during follow-up.

### Statistical analysis

The difference between groups was evaluated by independent samples *t* test. The level of significance was set as *P* < 0.05. All the statistical analysis was done with version 23.0 IBM SPSS statistics software.

## Results

**Fig. 1:**
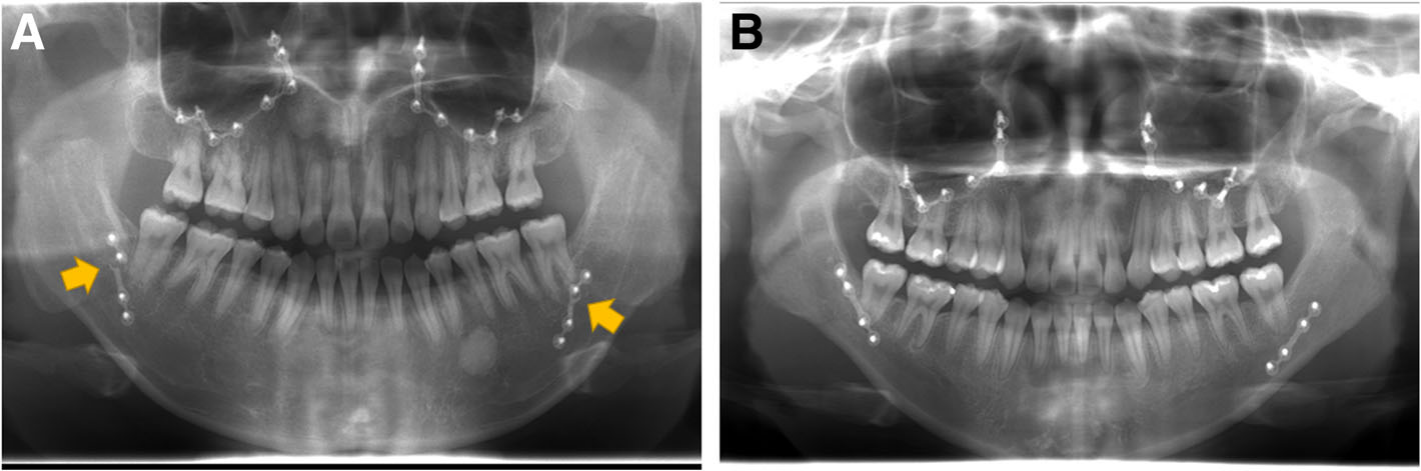
Panoramic view at 6 months after operation. **a** No treatment group showed plate fracture in both sides (arrows). **b** BTX-A injection group showed no plate fracture in both sides

**Fig. 2:**
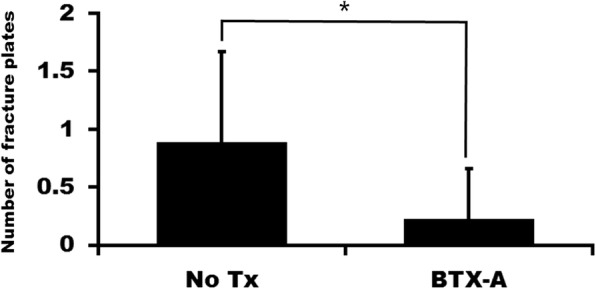
Number of fractured plates in each group (No Tx: no treatment group, BTX-A: BTX-A injection group, *P < 0.05)

The plate fracture was more frequently observed in the no treatment group than in the BTX-A injection group (Fig. 1). Total number of fractured plates in patients was 2 out of 16 plates in the BTX-A injection group and that was 8 out of 16 plates in the no treatment group. The average number of fractured plates in each patient was 1.00 ± 0.76 in the no treatment group and 0.25 ± 0.46 in the BTX-A injection group (Fig. 2). The difference between groups was statistically significant (*P* = 0.031).

Pre-operative SNB angle in the no treatment group was 82.36 ± 5.29° and that in BTX-A injection group was 86.25 ± 5.30° (Table 2). SNB angle at immediately after operation in no treatment group was 77.89 ± 4.90° and that in BTX-A injection group was 82.18 ± 3.79°. The differences between groups at pre-operation and immediately after operation were not significantly different (*P* > 0.05). SNB angle changes at follow-up compared to immediately after operation were 1.15 ± 1.18° and 0.24 ± 1.95° for the no treatment group and BTX-A injection group, respectively. Although no treatment group showed little higher change compared to the BTX-A injection group, the difference between groups were not significantly different (*P* > 0.05).

**Table 2:** Results of angular measurements

		No treatment	BTX-A injection	*P* value
SNB angle	Pre-operation	82.36 ± 5.29°	86.25 ± 5.30°	NS
	Immediately after operation	77.89 ± 4.90°	82.18 ± 3.79°	NS
	Post-operative change	1.15 ± 1.18°	0.24 ± 1.95°	NS
Mandibular plane angle	Pre-operation	34.03 ± 6.78°	32.01 ± 6.35°	NS
	Immediately after operation	32.97 ± 5.73°	31.99 ± 4.89°	NS
	Post-operative change	3.46 ± 3.89°	3.17 ± 3.37°	NS
Gonial angle	Pre-operation	125.06 ± 6.33°	130.93 ± 6.09°	NS
	Immediately after operation	122.83 ± 9.91°	126.12 ± 8.17°	NS
	Post-operative change	3.68 ± 4.08°	4.66 ± 3.07°	NS

*NS* not significant

Pre-operative mandibular plane angle in no treatment group was 34.03 ± 6.78° and that in BTX-A injection group was 32.01 ± 6.35° (Table 2). The mandibular plane angle was 32.97 ± 5.73° immediately after operation in the no treatment group, and in the BTX-A injection group, it was 31.99 ± 4.89°. The differences between groups at pre-operation and immediately after operation were not significantly different (*P* > 0.05). Mandibular plane angle changes at follow-up compared to immediately after operation was 3.46 ± 3.89° and 3.17 ± 3.37° for the no treatment group and BTX-A injection group, respectively (*P* > 0.05).

The pre-operative gonial angle in the no treatment group was 125.06 ± 6.33° and that in the BTX-A injection group was 130.93 ± 6.09° (Table 2). The gonial angle immediately after operation in the no treatment group was 122.83 ± 9.91°, and in the BTX-A injection group, it was 126.12 ± 8.17°. The differences between groups at pre-operation and immediately after operation were not significantly different (*P* > 0.05). Gonial angle changes at follow-up compared to immediately after operation were 3.68 ± 4.08° and 4.66 ± 3.07° for the no treatment group and the BTX-A injection group, respectively (*P* > 0.05).

## Discussion

BTX-A injection has been used for reduction of muscle power and pain. In this study, BTX-A injection into the masseter muscle was used for the prevention of post-operative complication after orthognathic surgery. The incidence of plate fracture was significantly lower in the BTX-A injection group than that in the no injection group (Fig. 2, *P* = 0.031). However, there was no statistically significant difference in the post-operative changes of SNB angle, mandibular plane angle, and gonial angle between groups (*P* > 0.05).

The BTX-A has been widely used for cosmetic purposes and therapeutic purposes in the peri-oral area and temporomandibular areas [4]. It has been used for the treatment of hemi-facial spasm, dystonia, hyperhidrosis, reducing wrinkles, and bruxism [1]. Some clinicians use BTX-A for controlling malocclusion after trauma [5]. Injection of BTX-A on the anterior belly of the digastric muscle showed favorable results on preventing relapse of anterior open bite after trauma [5]. BTX-A injection into the masticatory muscle in growing rats has shown changes in jaw bone morphology [21]. Based on these results, BTX-A injection has been used for preventing relapse after orthognathic surgery [22].

In this study, the incidence of plate fracture was shown to be significantly different between groups (Fig. 2). Although bicortical screws increase the stability of fixation after BSSRO, they increase the stress on the condyle [23]. Accordingly, mini-plate fixation is recommended for TMD patients [24]. As condylar position may influence post-operative occlusal stability, early removal of fixation is helpful for correcting occlusal discrepancies [25]. However, the mono-cortical absorbable plate system shows higher incidence of plate fracture compared to the bi-cortical plate fixation group [26]. When comparing the conventional absorbable plate system to the hybrid fixation system, plate fracture is observed only in the conventional absorbable plate system [27].

In this study, there was no significant difference between groups in post-operative changes of SNB angle, mandibular plane angle, and gonial angle (Table 2). The reasons could be explained as follows. First, some patients received genioplasty and/or LeFort I osteotomy as additional surgery. The number of each additional technique differed between groups (Table 1). As bone is an important site for muscle attachment, the difference in additional technique between groups might influence post-operative relapse. When Le Fort I osteotomy is combined with BSSRO, there are no differences in post-operative positional changes compared to BSSRO only [28]. However, mandibular angle or border reduction might influence post-operative positional stability. Second, any other muscles except for the masseter muscle can be involved in post-operative relapse. Therefore, masseter muscle-only injection might have a limited effect. Third, the number of samples was too small. Because of the small sample size, the patients could not be classified properly. Furthermore, in a study with a sufficient sample size, proper classification of the patients can show more accurate results.

Despite the significant difference in plate fractures between groups (Fig. 2), there was no difference in post-operative changes of angular measurement between groups (Table 2). Plate fractures were observed within 2 months post-operatively in this study (data not shown). As condylar position may influence on post-operative occlusal stability, early removal of fixation is helpful for correcting occlusal discrepancies [24]. Plate fracture might have similar effects to the removal of fixation. The mono-cortical fixation system shows similar stability to bicortical screw fixation after BSSRO [29]. In case of mandibular setback surgery, there are no differences in post-operative positional changes between bicortical plate fixation and monocortical plate fixation [30]. These findings show that post-operative relapse is a complicated situation which is influenced by multiple factors.

## Conclusions

BTX-A injection into the masseter muscle could reduce the incidence of plate facture. However, there was no statistically significant difference in post-operative changes of SNB angle, mandibular plane angle, and gonial angle between the BTX-A injection group and the no treatment group (*P* > 0.05).

## Abbreviation

BTX-A: Botulinum toxins, type A
